# Labor Induction in Women with Isolated Polyhydramnios at Term: A Multicenter Retrospective Cohort Analysis

**DOI:** 10.3390/jcm13051416

**Published:** 2024-02-29

**Authors:** Yael Lerner, Tzuria Peled, Morag Yehushua, Reut Rotem, Ari Weiss, Hen Y. Sela, Sorina Grisaru-Granovsky, Misgav Rottenstreich

**Affiliations:** 1 Department of Obstetrics & Gynecology, Shaare Zedek Medical Center, Faculty of Medicine, Affiliated with the Hebrew University School of Medicine, Jerusalem 91031, Israel; 2 Department of Nursing, Jerusalem College of Technology, Jerusalem 9548301, Israel

**Keywords:** expectant management, labor induction, polyhydramnios, perinatal outcomes, term pregnancy, obstetric interventions, maternal morbidity, neonatal morbidity, uterine rupture, fetal death, fetal distress, gestational diabetes, composite maternal outcome, elective induction, multicenter study, low risk

## Abstract

**Background:** With the increasing popularity of elective induction after 39 + 0 weeks, the question of whether induction of labor (IOL) is safe in women with isolated polyhydramnios has become more relevant. We aimed to evaluate the pregnancy outcomes associated with IOL among women with and without isolated polyhydramnios. **Methods:** This was a multicenter retrospective cohort that included women who underwent induction of labor at term. The study compared women who underwent IOL due to isolated polyhydramnios to low-risk women who underwent elective IOL due to gestational age only. The main outcome measure was a composite adverse maternal outcome, while the secondary outcomes included maternal and neonatal adverse pregnancy outcomes. **Results:** During the study period, 1004 women underwent IOL at term and met inclusion and exclusion criteria; 162 had isolated polyhydramnios, and 842 had a normal amount of amniotic fluid. Women who had isolated polyhydramnios had higher rates of the composite adverse maternal outcome (28.7% vs. 20.4%, *p* = 0.02), prolonged hospital stay, perineal tear grade 3/4, postpartum hemorrhage, and neonatal hypoglycemia. Multivariate analyses revealed that among women with IOL, polyhydramnios was significantly associated with adverse composite maternal outcome [aOR 1.98 (1.27–3.10), *p* < 0.01]. **Conclusions:** IOL in women with isolated polyhydramnios at term was associated with worse perinatal outcomes compared to low-risk women who underwent elective IOL. Our findings suggest that the management of women with polyhydramnios cannot be extrapolated from studies of low-risk populations and that clinical decision-making should take into account the individual patient’s risk factors and preferences.

## 1. Introduction

Qualitative or subjective assessment of amniotic fluid volume is a standard component of any obstetric ultrasound examination, as it is considered an indicator of fetoplacental activity or as a warning sign for underlying fetal, maternal, or placental abnormality. Thus, the amniotic fluid amount measurement is useful for identifying, monitoring, and managing complicated pregnancies [1].

Polyhydramnios (PH) is defined as a pathological increased amniotic fluid amount during pregnancy [2], reflected as a single deepest vertical pocket (DVP) of amniotic fluid >8 cm or an amniotic fluid index (AFI) > 24 cm [1]. It has been accepted to classify PH into three groups according to severity. Mild PH (AFI of 25–30 cm or deepest amniotic fluid pocket 3–8 cm), moderate PH (AFI 30.1–35 cm or deepest amniotic fluid pocket 12–15 cm), and severe PH (AFI ≥  35.1 cm or deepest amniotic fluid pocket ≥ 16 cm) [2]. The reported incidence of PH, irrespective of its etiology, varies between 1 and 2% in various studies [3,4].

PH can be caused by a variety of etiologies and pregnancy complications; the most common causes include pre-gestational and gestational diabetes, fetal anomalies with disturbed fetal swallowing, and fetal infections [1,2]. Idiopathic or isolated PH refers to instances where PH occurs without an apparent pathological context, such as congenital anomalies, genetic abnormalities, maternal diabetes mellitus, fetal infections, placental tumors, or multiple gestations, constituting approximately 50–60% of PH cases [5]. Notably, in 10% of cases initially classified as isolated PH, an abnormality is later diagnosed postnatally [6].

PH is associated with an elevated risk of adverse maternal and neonatal outcomes regardless of its etiology, most probably due to overdistension of the uterus. These complications include maternal dyspnea, urinary tract infection, hypertensive disorders of pregnancy, preterm labor, premature rupture of membranes, abnormal fetal presentation, umbilical cord prolapse, uterine rupture, fetal macrosomia, and postpartum hemorrhage [2,7,8,9,10], as well as an increase in cesarean deliveries [11]. In addition, in non-anomalous pregnancies affected by PH, the risk of intrauterine fetal death is greater at every gestational age, with the greatest increase in risk at term. At 40 weeks, the risk for intrauterine fetal death escalates by 11-fold, compared to pregnancies with no such diagnosis [12]. The maternal and neonatal prognosis in pregnancies affected by PH is known to depend on the degree of severity and the underlying etiology [1,2].

Due to the complications found to be associated with PH and the increased maternal and neonatal risks, there arises the important question regarding the preferable mode and the optimal timing of delivery. Guidelines from the Society for Maternal-Fetal Medicine (SMFM) propose allowing spontaneous onset of labor at term for women with mild isolated PH [1]. In contrast, the American College of Obstetricians and Gynecologists (ACOG) recommends delivery between 39 + 0 and 40 + 6 weeks in those cases [13]. However, the data regarding this issue are inconclusive and scant, and there is no well-established study regarding induction of labor outcomes in women with PH.

In general, recommendations regarding the timing of delivery are founded on a balancing of maternal and perinatal risks. Induction of labor is indicated when continuation of the pregnancy is thought to be associated with maternal or neonatal increased risks.

In 2018, Grobman et al. published the “ARRIVE” trial, which had a great impact on physician decisions regarding induction of labor. The study aimed to determine whether elective induction of labor improves maternal and neonatal outcomes. The study was conducted on a cohort of low-risk nulliparous women with a singleton, vertex uncomplicated term pregnancy at 39 weeks of gestation. They compared maternal and neonatal outcomes of induction of labor versus expectant management. The study demonstrated that elective induction in this subgroup of low-risk nulliparous women resulted in a significantly lower frequency of cesarean deliveries but with no significantly lower frequency of composite adverse perinatal outcomes [14] 

Since the publication of the ARRIVE trial, which demonstrated the safety of induction of labor at 39 weeks in low-risk pregnancies and its probable advantage, many obstetric organizations and guidelines published a response, and further studies were conducted. The SMFM and the ACOG acknowledged that it may be reasonable to offer nulliparous women the option of an elective induction of labor at 39 weeks of gestation [15,16]. And additional studies supported that induction of labor might decrease perinatal deaths, cesarean deliveries, and neonatal intensive care unit (NICU) admissions compared with expectant management [16,17]. Even though they did not endorse practice policy change, the frequency of induction of labor is increasing, and more women and physicians are considering induction of labor at term, even without indication [18,19,20]. Regarding induction of labor in other population groups of women, there is evidence of benefits in induction of labor in multiparous low-risk women [21] and even at nulliparous with advanced maternal age [22].

However, the advantages of induction of labor were demonstrated in those studies only in low-risk and uncomplicated pregnancies, and it is clear that additional studies are needed to prove the safety and advantages of induction of labor, also in other subpopulations groups of women that are not at low risk [19]. 

Specifically, data regarding the risks or benefits of induction of labor for women with pregnancies affected by PH at term are limited, and it is not clear whether it is safe and improves perinatal outcomes.

Therefore, this study aims to evaluate the maternal and neonatal outcomes associated with induction of labor at term for women with and without isolated PH.

## 2. Materials and Methods

### 2.1. Study Design

Our investigation constituted a multicenter retrospective cohort analysis utilizing computerized medical records sourced from two university-affiliated obstetric centers located in Jerusalem, Israel, Shaare Zedek Medical Center (SZMC) and Bikur Holim Medical Center (BHMC). 

### 2.2. Study Population and Period

These centers deliver obstetric care to a population exceeding 1,200,000 residents and contribute to approximately 16% of all deliveries in Israel, with an annual average of 22,000 deliveries. The medical records undergo real-time updates during labor, delivery, and surgery by attending healthcare personnel. Regular audits conducted by trained technical personnel ensure data accuracy and mitigate potential biases inherent in retrospective studies.

### 2.3. Inclusion and Exclusion Criteria

The study encompassed women with a singleton pregnancy of a live fetus who underwent induction of labor between 39 + 0 and 41 + 6 gestational weeks from 2016 to 2021 in our centers. Exclusion criteria comprised the following: (1) high-risk women with maternal or fetal indications for induction of labor other than PH (e.g., maternal morbidities, hypertensive disorders, placental complications, suspected fetal distress, decreased fetal movements, or non-reassuring fetal heart rate), (2) women with previous cesarean deliveries, (3) multifetal gestation, (4) non-isolated PH (pre-gestational or gestational diabetes, fetal major malformations, fetal known genetic abnormalities, fetal infections, or placental tumors), (5) previous 3/4 grade perineal tear, (6) pre-labor fetal death, (7) planned cesarean delivery, (8) non-vertex presentation, and (9) out-of-hospital deliveries.

### 2.4. Comparison Groups

Women subjected to induction of labor due to isolated PH were compared to a control group of low-risk women who underwent elective induction of labor without maternal or neonatal indications other than gestational age. 

### 2.5. Definitions

PH was defined by either (1) a single deepest vertical pocket (DVP) of amniotic fluid > 8 cm or (2) an amniotic fluid index (AFI) > 24 cm. The degree of severity of PH was categorized as mild, moderate, or severe based on an AFI of 24.0–29.9 cm, 30.0–34.9 cm, and ≥35 cm or a DVP of 8–11 cm, 12–15 cm, or ≥16 cm, respectively [1,2]. All women included in the study underwent a full biophysical profile assessment (ultrasound and non-stress test) upon admission to Labor and Delivery.

Following departmental protocol, induction of labor was recommended at 39 weeks gestational age for women with isolated PH.

### 2.6. Outcomes Measures

The primary outcome was a composite adverse maternal outcome, defined by the occurrence of at least one of the following: cesarean delivery, postpartum hemorrhage (estimated blood loss > 1000 mL and/or hemoglobin drop ≥ 3 g/dL), blood products transfusion, severe perineal tear 3/4 grade, uterine rupture, laparotomy, hysterectomy, maternal Intensive Care Unit admissions, and prolonged hospitalization (≥7 days after cesarean > 5 days after vaginal delivery). Secondary outcomes included maternal and neonatal adverse pregnancy outcomes.

Maternal morbidity was assessed by analyzing parameters such as postpartum hemorrhage (estimated blood loss > 1000 mL and/or hemoglobin drop ≥ 3 g/dL), blood products transfusion, chorioamnionitis, severe perineal tear 3/4 grade, uterine rupture, laparotomy, hysterectomy, puerperal fever, maternal Intensive Care Unit admissions, and prolonged hospitalization (≥7 days after cesarean > 5 days after vaginal delivery). Neonatal morbidity was assessed by analyzing the occurrence of intrapartum fetal death, 5 min Apgar score < 7, NICU admission, neonatal asphyxia, meconium aspiration, jaundice, transient tachypnea of the newborn, brachial plexus injury, mechanical ventilation, convulsions, hypoglycemia, sepsis, encephalopathy, and intracranial hemorrhage.

### 2.7. Ethical Considerations

The study adhered to the ethical principles of the Declaration of Helsinki and received approval from the Institutional Review Board of the medical centers (IRB approval number: 0270-22, date of approval—18 October 2022). De-identified data from medical records were used, and patient participation was not required; hence, written informed consent was not obtained.

### 2.8. Statistical Methods

Statistical methods involved descriptive statistics, including proportions for nominal variables, means ± standard deviation for continuous variables with normal distribution, and medians with interquartile ranges (IQR) for continuous variables without normal distribution. Categorical variables were compared using the Chi-square test or Fisher’s exact test, and continuous variables were analyzed using the unpaired Student’s *t*-test or Mann–Whitney test. A *p*-value < 0.05 was considered statistically significant for all tests.

Univariate analyses were conducted to compare outcomes between women with isolated PH and those without. Variables found significant on univariate analysis for the composite adverse maternal outcome were included in a multivariable logistic regression model. These variables included maternal age, previous miscarriages, parity, fertility treatments, smoking, cervical dilation on admission, cervical ripening, oxytocin augmentation of labor, and epidural analgesia. The association between PH and the composite adverse maternal outcome was modeled using adjusted odds ratios (aOR) with 95% confidence intervals (CIs). All statistical tests were two-sided, and analyses were performed using SPSS software (version 25 statistical package: IBM, Armonk, NY, USA).

## 3. Results

**Figure 1:**
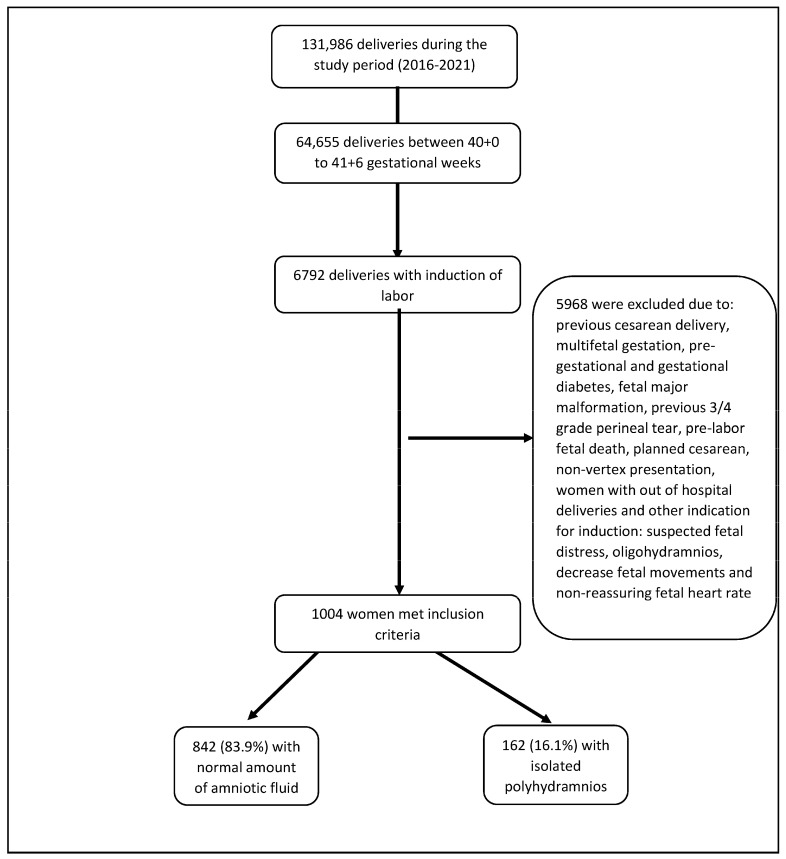
Labor analyze.

Throughout the study period, 1004 women underwent induction of labor between 39 + 0 and 41 + 6 weeks of gestation, meeting the predetermined inclusion and exclusion criteria. Among them, 162 underwent induction of labor due to isolated PH, while 842 underwent elective induction of labor with a normal amount of amniotic fluid (Figure 1). Of those with isolated PH, 86 (53.1%) had mild, 61 (37.7%) had moderate, and 15 (9.3%) had severe PH. 

Table 1 presents the maternal demographic, obstetric, and neonatal characteristics, comparing women with pregnancies affected by isolated PH who underwent induction of labor at term to women with normal amniotic fluid amount who underwent elective induction of labor due to gestational age only. Overall, women who underwent induction of labor due to isolated PH exhibited similar characteristics to those opting for elective induction of labor, except for higher rates of obesity (43 [32.3%] vs. 140 [21%], *p* < 0.01) and a statistically significant earlier gestational age at delivery (40.4 ± 0.5 vs. 40.5 ± 0.5, *p* = 0.01).

Maternal obstetric outcomes are detailed in Table 2. Women with isolated PH demonstrated higher rates of postpartum hemorrhage (16.7% vs. 11%, *p* = 0.04), perineal tear grade 3/4 (1.7% vs. 0.4%, *p* = 0.03), and prolonged hospital stay (5.7% vs. 2.6%, *p* = 0.03). Notably, there was one case of intrapartum fetal death following uterine rupture, diagnosed immediately after labor via laparotomy in this group. The composite adverse maternal outcome was significantly higher among women with pregnancies affected by isolated PH (28.7% vs. 20.4%, *p* = 0.02).

Table 3 presents neonatal outcomes comparing women with pregnancies affected by isolated PH who underwent induction of labor at term to women with normal amniotic fluid amount who underwent elective induction of labor due to gestational age only. The mean neonatal birth weight was significantly higher in the group of women with pregnancies affected by isolated PH (3788.6 ± 369.5 vs. 3594.1 ± 434.1, *p* < 0.01), along with higher rates of macrosomia (21.3% vs. 11.3%, *p* < 0.01), male gender (62.1% vs. 50.8%, *p* = 0.01), and hypoglycemia (3.4% vs. 0.8%, *p* < 0.01). Multivariate analyses in Table 4 revealed that among women undergoing induction of labor at term, isolated PH was independently associated with a composite adverse maternal outcome [adjusted odds ratio (aOR) 1.98 (1.27–3.10), *p* < 0.01].

## 4. Discussion

In this retrospective multicenter study comprising 1004 women undergoing induction of labor at term, we observed that 162 women had induction of labor due to isolated PH, while 842 women underwent elective induction of labor with normal amniotic fluid levels. Notably, women with pregnancies affected by PH exhibited unfavorable maternal and perinatal outcomes compared to women who underwent elective induction of labor, including elevated rates of postpartum hemorrhage, high-degree perineal tears, prolonged hospital stays, and neonatal hypoglycemia. Furthermore, a case of uterine rupture and fetal death was documented in the PH group. Multivariate analysis substantiated these findings, highlighting a significant association between PH and a composite adverse maternal outcome.

We demonstrated a relatively high prevalence of PH in our study (162/1004, 16%), then observed in previous studies [3,4]. This stems from meticulous case selection and stringent exclusion criteria. Our control group comprised only low-risk cases of women who underwent elective induction of labor with no additional maternal or neonatal indications, except for gestational age—a control group that constitutes a relatively small percentage of our population. Consequently, the true incidence of PH in our population cannot be accurately determined from this study and is likely lower.In alignment with prior research, our study contributes to the growing body of evidence that establishes a correlation between PH and adverse maternal and neonatal outcomes. The concept of uterine overdistension in pregnancies affected by PH, as highlighted by Hamza et al., has been consistently associated with an elevated risk of various complications, including postpartum hemorrhage, cord prolapse, and malpresentation [2]. Several comprehensive studies by Pilliod et al. [12], Karahanoglu et al. [7], Zeino et al. [9], and Vanda et al. [23] underscore the heightened other maternal and neonatal risks associated with PH, encompassing macrosomia, induction of labor, prolonged labor, cesarean delivery, non-vertex presentation, intrauterine fetal death, and respiratory distress syndrome, among other complications. An important study in this issue, conducted by Pilloid et al., aimed to evaluate the ongoing risk of intrauterine fetal death in isolated PH pregnancies affected by isolated PH. They demonstrated a greater risk of intrauterine fetal death in isolated PH pregnancies at every gestational age compared with pregnancies with normal amniotic fluid amounts. The risk increased gradually from a 7-fold higher risk than the control group at 37 weeks of gestation up to a 11-fold higher risk at 40 weeks of gestation. This association also remained statistically significant after adjusting for multiple confounding variables was conducted [12].

While existing guidelines recommend induction of labor at term for isolated PH mainly to reduce the risk of stillbirth, the optimal timing remains debated due to the lack of prospective or retrospective studies comparing induction to expectant management in these cases. Recent studies have noted a rise in induction rates in isolated PH pregnancies without other indications, raising questions about the safety and outcomes of induction in these high-risk scenarios [1,13].

In recent years, there has been a growing body of evidence to support elective induction after 39 + 0 weeks for women with low-risk pregnancies [14], following data regarding the safety of induction of labor, the popularity of induction of labor in high-risk scenarios, including PH also increased [24]. Therefore, the question of whether induction of labor is safe in high-risk women or high-risk pregnancies has become more relevant [14,25,26,27]. Recent studies have shown a statistically significant increase in the rate of induction of labor in isolated PH pregnancies without any other indication for induction [5,9,28]. Bas Lando et al. demonstrated that isolated PH at term is independently associated with worse outcomes during and after labor, including fetal macrosomia, cesarean deliveries, and neonatal complications, regardless of whether it was spontaneous onset of labor or induction of labor [11]. So, despite the compelling evidence of worse outcomes observed in our study among women with isolated PH, the specific causal factor that impacts these unfavorable outcomes attributed to the PH itself or the induction of labor in cases of PH remains unclear. This uncertainty underscores the importance of carefully considering induction of labor policies and their associated outcomes when advising and consulting women with PH. Addressing these complexities is vital in providing nuanced and evidence-based recommendations for the management of pregnancies affected by PH, particularly in the context of the contemporary landscape where elective induction practices are gaining traction.

Following the work of Pilliod et al. described above, which demonstrated the increased risk of intrauterine fetal death in PH pregnancies [12], there has been ongoing debate regarding the optimal timing and mode of delivery for this subpopulation. While it is common practice to consider induction of labor at term in PH pregnancies, no well-established prospective studies have definitively shown that induction of labor is preferable to expectant management in terms of outcomes. One retrospective study by Backely et al. compared outcomes in PH cases, with 97 women undergoing induction of labor between 38 0/7 and 39 6/7 weeks and 71 women undergoing expectant management. While they found that induction of labor reduced the risk of cesarean delivery, there were no significant differences in other maternal or neonatal outcomes between the two groups. However, there was a case of hysterectomy and a case of cord prolapse in the induction of labor group, while the expectant management group had one case of Intrauterine fetal death due to placental insufficiency [29]. Therefore, it is not possible to conclude from this study which approach is safer or preferable—induction of labor or expectant management—and further research is needed to clarify this issue.

This study focused solely on women with isolated PH while excluding any other etiology of PH. All women in the study group underwent a screening test for gestational diabetes mellitus (50 g oral glucose load between 24 and 28 weeks of gestation), and those with pre-gestational, gestational diabetes mellitus, or unknown diabetes status were excluded. Despite this exclusion, the rates of macrosomic neonates (21.3% vs. 11.3%, *p* < 0.01) and neonatal hypoglycemia (3.4% vs. 0.8%, *p* < 0.01) were significantly higher among women with PH compared to women with a normal amount of amniotic fluid, while the association between isolated PH and macrosomia has been shown before [11,30,31]. This raises the question of whether there was a missed diagnosis of gestational diabetes mellitus in the PH group that could also have affected maternal and neonatal outcomes. Therefore, the possibility of gestational diabetes mellitus in women with negative screening should be considered during the management of women with PH. While the SMFM guidelines suggest considering rescreening for gestational diabetes mellitus when PH is identified in the third trimester and/or >1 month has elapsed since diabetes screening was completed [1], there are no data to support that recommendation for women with PH. Interestingly, in a study from the UK, when gestational diabetes mellitus screening was performed for women with risk factors in the second trimester using a 75 g OGTT, the role of a rescreening after 33 gestational weeks for women with new risk factors such as glycosuria, and estimated fetal weight or abdominal circumference > 95th centile on ultrasound scan was assessed, 46.0% were diagnosed as having gestational diabetes mellitus in the third trimester and targeted intervention was associated with better obstetric and neonatal outcomes [32]. Future studies should evaluate the role of rescreening vs. diagnostic tests for gestational diabetes mellitus among women with isolated PH. However, rare etiologies such as Costello syndrome should also be considered [33].

Our study is unique as it specifically focuses on the maternal and neonatal outcomes of induction of labor in women with isolated PH, without any other maternal or neonatal concerns, and compares them to a “clean” control group of women in low-risk pregnancy. This study provides crucial information on induction of labor outcomes in this specific subpopulation and can serve as a basis for re-evaluating the current timing and mode of delivery policies for women with isolated PH.

However, since the practical dilemma is whether to recommend induction of labor for women with PH, it is crucial to acknowledge that our study primarily focused on the exposure being PH rather than induction of labor. As isolated PH is independently associated with worse perinatal outcomes, it is not clear whether induction of labor itself worsens those outcomes [2,11]. Therefore, our study does not provide a direct answer to the question of what course of action to take in terms of offering induction of labor. The nature of our investigation is purely scientific, emphasizing the association between PH and adverse outcomes. Hence, this study draws the attention of physicians to the fact that women with pregnancies affected by PH are at higher risk during induction of labor and that caution should be taken into account.

Given the potential legal implications, it is imperative to recognize that this type of data may raise legal concerns. Consequently, we advocate for prospective studies that specifically compare induction of labor versus expectant management in term pregnancies with isolated PH. Such studies might provide more comprehensive insights into the risks and benefits associated with different management strategies in this high-risk population. Then, valuable guidance for clinical decision making and potentially addressing legal considerations might be suggested.

### Strengths and Limitations

The study’s main strength is that being a multicenter study with high annual delivery volumes in a developed country with advanced medical services enables us to examine a relatively large cohort of women with isolated PH and compare them to a low-risk group of women. Besides that, there are several additional notable strengths of this study: (1) this study isolated the differences associated with PH only by comparing labor induction of women with PH to elective induction of labor with no other maternal and neonatal indication for induction besides gestational age; (2) real-time data validation was performed, providing comprehensive data and minimized bias; (3) all costs of antenatal care, birth, postpartum care for mother and child were uniformly covered by National Health Insurance for the entire study period, which may limit possible medical treatment disparities among different women; (4) all mother–child data included were taken from a singular hospital setting with no inter-hospital transfers, which may overcome potential selection bias; and (5) extensive retrospective data extraction resulted in two overall similar study groups.

All these study advantages mentioned above strengthen the validity of our findings, reduce the risk of biases and confounders, and increase the generalizability to a wider range of women.

However, several limitations to this study should be considered: (1) The inherent limitation of our study’s retrospective design, which is based on data extracted from women’s medical records; (2) The relatively small study groups may preclude generalization of the study and did not enable us to stratify the complications to the severity degree of PH. Nonetheless, the multicenter design may attest to the robustness of the data and valid interpretation of the results; (3) The most significant limitation is that we did not examine the real clinical dilemma of whether induction of labor of women with PH has a better outcome than expectant management; and (4) the study includes women with different degrees of PH, and the sample size of the study was too small to assess the outcomes in the different subgroups.

## 5. Conclusions

Our study revealed that inducing labor in women with pregnancies affected by PH at or above 39 0/7 weeks of gestation, when compared to elective induction of labor in low-risk women with a normal amniotic fluid amount, is associated with an increased risk of adverse maternal and neonatal outcomes. Our findings suggest that the management of women with pregnancies affected by PH cannot be extrapolated from studies of low-risk populations and that clinical decision making regarding induction of labor should take into account the individual patient’s risk factors and preferences. Future real-time prospective studies comparing induction of labor with expectant management in pregnancies affected by PH at term are needed to gain a deeper understanding of this topic. These studies should also take into account the effect of the different amounts of amniotic fluid on the adverse outcomes.

## Figures and Tables

**Table 1:** Demographic and obstetric characteristics of women with IOL due to isolated polyhydramnios and elective IOL.

	Normal Amount of Amniotic Fluid n = 842	Polyhydramnios n = 162	*p* Value
Maternal age, years	30.1 ± 6.2	30 ± 6.4	0.90
Miscarriages, any	277 (32.9%)	63 (36.2%)	0.39
Miscarriages ≥ 3	49 (5.8%)	14 (8%)	0.27
Gravidity	4 [2–6]	4 [2–7]	0.11
Parity	3 [1–5]	3 [2–6]	0.10
Primiparity	232 (27.5%)	41 (23.6%)	0.28
Fertility Treatments	50 (5.9%)	12 (6.9%)	0.63
Hypertensive disorders of pregnancy	5 (0.6%)	1 (0.6%)	0.98
Smoking	16 (2.1%)	3 (1.8%)	0.85
Obesity (BMI ≥ 30)	140 (21%)	43 (32.3%)	<0.01
Anemia, Hb < 11 g/dL on admission	109 (12.9%)	30 (17.2%)	0.13
Cervical dilation on admission	2 [1–3]	2 [1–3]	0.09
Cervical ripening	260 (32.4%)	40 (25.5%)	0.09
Meconium-stained amniotic fluid	136 (16.1%)	31 (17.8%)	0.59
Epidural analgesia	732 (86.8%)	152 (87.4%)	0.85
Persistent Occipito-Posterior	21 (3.5%)	7 (5.8%)	0.22
Gestational age at delivery	40.5 ± 0.5	40.4 ± 0.5	<0.01

Data are mean ± standard deviation; median [interquartile range], number (%).

**Table 2:** Obstetric maternal outcomes among the study groups.

	Normal Amount of Amniotic Fluid n = 842	Polyhydramnios n = 162	*p* Value
**Primary outcome**			
Composite adverse maternal outcome *	172 (20.4%)	50 (28.7%)	0.02
**Components of the primary outcome**			
Unplanned cesarean	79 (9.4%)	19 (10.9%)	0.53
Postpartum hemorrhage	93 (11%)	29 (16.7%)	0.04
Blood products transfusion	7 (0.8%)	4 (2.3%)	0.09
Chorioamnionitis	45 (5.3%)	5 (2.9%)	0.17
Perineal tear grade 3/4	3 (0.4%)	3 (1.7%)	0.03
Uterine rupture	0 (0%)	1 (0.6%)	0.03
Hysterectomy	0 (0%)	0 (0%)	N/A
Laparotomy	0 (0%)	1 (0.6%)	0.03
Puerperal fever	34 (4%)	4 (2.3%)	0.27
Maternal ICU admissions	0 (0%)	0 (0%)	N/A
Prolonged hospital stays	22 (2.6%)	10 (5.7%)	0.03
**Other outcomes**			
Gestational age at delivery > 41 week	269 (31.9%)	49 (28.2%)	0.33
First stage duration, minutes	555.4 ± 382.2	591.7 ± 430.5	0.31
Second stage duration, minutes	39.9 ± 57.5	42.9 ± 62	0.56
Episiotomy	106 (12.6%)	18 (10.3%)	0.41
Shoulder dystocia	6 (0.7%)	2 (1.1%)	0.55
Retained placenta/placental fragments	31 (3.8%)	11 (6.5%)	0.12
Hemoglobin drop, g/dL	1.3 ± 1.1	1.5 ± 1.3	0.20
Hemoglobin drop > 4 g/dL	33 (3.9%)	12 (6.9%)	0.08
Vacuum-assisted delivery	83 (9.8%)	19 (10.9%)	0.67
Hospitalization length, days	2.3 ± 1.3	2.8 ± 2.4	<0.01

Data are mean ± standard deviation; number (%); ICU, Intensive Care Unit. * A composite adverse maternal outcome including at least one of the following: postpartum hemorrhage (estimated blood loss > 1000 mL and/or hemoglobin drop ≥ 3 g/dL), blood products transfusion, chorioamnionitis, severe perineal tear 3/4 grade, uterine rupture, laparotomy, hysterectomy, puerperal fever, maternal Intensive Care Unit admissions, and prolonged hospitalization (≥7 days after cesarean >5 days after vaginal delivery).

**Table 3:** Obstetric neonatal outcomes among the study groups.

	Normal Amount of Amniotic Fluid n = 842	Polyhydramnios n = 162	*p* Value
Birthweight	3594.1 ± 434.1	3788.6 ± 369.5	<0.01
Birthweight ≥ 4000 g	95 (11.3%)	37 (21.3%)	<0.01
LGA	143 (17%)	59 (33.9%)	<0.01
Male sex	428 (50.8%)	108 (62.1%)	<0.01
1-Minute Apgar score *<* 7	30 (3.6%)	6 (3.4%)	0.94
5-Minute Apgar score *<* 7	6 (0.7%)	2 (1.1%)	0.55
Intrapartum Fetal Death	0 (0%)	1 (0.6%)	0.03
NICU admission	24 (2.8%)	7 (4%)	0.41
Meconium aspiration syndrome	1 (0.1%)	0 (0%)	0.65
Jaundice	49 (5.8%)	8 (4.6%)	0.52
TTN	8 (1%)	1 (0.6%)	0.63
Mechanical ventilation	13 (1.5%)	2 (1.1%)	0.69
Seizures	11 (1.3%)	6 (3.4%)	0.05
Erb’s palsy/fracture of clavicle	5 (0.6%)	1 (0.6%)	0.98
Hypoglycemia	7 (0.8%)	6 (3.4%)	<0.01
Sepsis	3 (0.4%)	0 (0%)	0.43
Encephalopathy	1 (0.1%)	0 (0%)	0.65
Intracranial hemorrhage	4 (0.5%)	0 (0%)	0.36
Birth asphyxia	4 (0.5%)	2 (1.1%)	0.29

Data are mean ± standard deviation; number (%); LGA, large for gestational age, NICU, neonatal intensive care unit, TTN, transient tachypnea of the newborn.

**Table 4:** Multivariate logistic regression analysis for the association between polyhydramnios and composite adverse maternal outcome * (adjusted odds ratio).

	*p* Value	aOR	95%CI	
Parity	<0.01	0.72	0.65	0.81
Polyhydramnios	<0.01	1.98	1.27	3.10
Epidural analgesia	0.01	0.50	0.31	0.82
Maternal age, years	0.01	1.05	1.01	1.08

CI, confidence interval, aOR, adjusted odds ratio. * A composite adverse maternal outcome including at least one of the following: preterm delivery at <37 0/7 weeks, postpartum hemorrhage, blood products transfusion, maternal ICU admissions, prolonged hospitalization (≥7 days after cesarean > 5 days after vaginal delivery), uterine rupture, laparotomy, and hysterectomy.

## Data Availability

Due to the privacy concerns surrounding patient data, the data are not available for sharing.
